# Debye-Hückel Free Energy of an Electric Double Layer with Discrete Charges Located at a Dielectric Interface

**DOI:** 10.3390/membranes11020129

**Published:** 2021-02-14

**Authors:** Guilherme Volpe Bossa, Sylvio May

**Affiliations:** 1 Department of Physics, Institute of Biosciences, Humanities and Exact Sciences, São Paulo State University (UNESP), São José do Rio Preto 15054-000, Brazil; guilherme.vbossa@outlook.com; 2 Department of Physics, North Dakota State University, Fargo North Dakota, ND 58108-6050, USA

**Keywords:** Fourier-Bessel sum, dielectric interface, Debye-Hückel, electrostatics, screened Coulomb potential, dipole interactions

## Abstract

Poisson–Boltzmann theory provides an established framework to calculate properties and free energies of an electric double layer, especially for simple geometries and interfaces that carry continuous charge densities. At sufficiently small length scales, however, the discreteness of the surface charges cannot be neglected. We consider a planar dielectric interface that separates a salt-containing aqueous phase from a medium of low dielectric constant and carries discrete surface charges of fixed density. Within the linear Debye-Hückel limit of Poisson–Boltzmann theory, we calculate the surface potential inside a Wigner–Seitz cell that is produced by all surface charges outside the cell using a Fourier-Bessel series and a Hankel transformation. From the surface potential, we obtain the Debye-Hückel free energy of the electric double layer, which we compare with the corresponding expression in the continuum limit. Differences arise for sufficiently small charge densities, where we show that the dominating interaction is dipolar, arising from the dipoles formed by the surface charges and associated counterions. This interaction propagates through the medium of a low dielectric constant and alters the continuum power of two dependence of the free energy on the surface charge density to a power of 2.5 law.

## 1. Introduction

Charged interfaces in aqueous solution give rise to the formation of an electric double layer—a diffuse cloud of co- and counter-ions that screen the interfacial charges [1,2]. Electric double layers are abundant in every living cell, as well as in many technological applications. Among the most prominent examples are biomembranes, which consist of a mixture of lipids and associated proteins that self-assemble into an extended planar sheet. Some of the lipids are usually anionic, with their headgroup charges being located at the dielectric interface between a region of low dielectric constant inside the membrane and the aqueous phase, which has a large dielectric constant and contains salt ions [3].

The properties of the electric double layer can be described at different levels of abstraction, varying from detailed atomistic and coarse-grained computer simulations [4] to the most simple mean-field models. A major feature of the latter is the neglect of ion-ion correlations [5]. Mean-field models become especially useful [6,7] and amenable to extensions [8,9] when based on simple geometries and when treating the dielectric interface as uniformly charged. One of the most important extensions of applying mean-field electrostatics to the electric double layer involves inhomogeneously charged surfaces, ranging from continuous charge distributions to discrete point charges. Surfaces with patchy or non-uniform charge distributions [10,11,12,13], domain formation and phase separations [14,15], and domain boundaries [16,17,18] all have been modeled using mean-field electrostatics while maintaining the assumption of charge continuity, which is justified on sufficiently large length scales. For weakly charged surfaces or on sufficiently small length scales, the discreteness of the electric charge will become noticeable. This has inspired a number of authors to compute the electrostatic potential produced by an ordered array of surface charges [19,20,21] or even a single surface charge [22,23,24,25] and others to use virial expansions [26,27] and Monte Carlo simulations [28,29,30,31,32,33,34,35,36,37] to account for charge discreteness. Some studies have focused on the pressure that acts across electrolytes due to the presence of discrete charges [28,35,38], yet—perhaps somewhat surprisingly—there has not been an attempt so far to compute the free energy of a single planar dielectric interface with discrete charges and compare this with the corresponding free energy derived for a continuous charge distribution. Filling this gap on the level of the linearized Debye-Hückel model is the goal of the present work.

We study the properties of an electric double layer on the basis of the linearized Debye-Hückel limit, which becomes valid for a sufficiently small average surface charge density. Discrete charges are attached with a certain density to a planar dielectric interface that separates a salt-containing aqueous solution from a medium with a low dielectric constant. We introduce a Wigner–Seitz cell model [39] to account for the interactions between the interfacial charges, which do not need to form an ordered array. Using a Fourier-Bessel series and a Hankel transformation, we calculate the electrostatic surface potential at the position of an individual charge produced by all other charges on the interface and, from that, the Debye-Hückel free energy of the electric double layer. The discrete nature of the interfacial charges becomes important for sufficiently small densities. In this case, the interactions between the interfacial charges are dominated by dipolar contributions mediated through the salt-free medium of low dielectric constant.

## 2. Theory and Discussion

We consider a flat interface of sufficiently large lateral area *A* that separates two distinct dielectric media. One medium has a dielectric constant $\varepsilon_{l}$, and the other medium has a dielectric constant $\varepsilon_{w}$ and contains monovalent anions and cations of a salt such as NaCl, both of bulk concentration $n_{0}$. Because a lipid layer is representative of the systems we intend to model, we chose the subscripts “l” and “w” for the “lipid” hydrocarbon core and “water”, respectively, where $2≲\varepsilon_{l}≲4$ and $\varepsilon_{w}\approx 80$. However, our theoretical model is general and applies to any choices of $\varepsilon_{l}$ and $\varepsilon_{w}$. The presence of salt ions entails the formation of an electric double layer in the medium of dielectric constant $\varepsilon_{w}$. The characteristic screening length of the electric double layer is given by the Debye length $l_{D}={\left[\left(\varepsilon_{w}\varepsilon_{0}k_{B}T\right)/\left(2e^{2}n_{0}\right)\right]}^{1/2}$, where $\varepsilon_{0}$ is the vacuum permittivity, $k_{B}$ Boltzmann’s constant, *T* the absolute temperature, and *e* the elementary charge.

The dielectric interface also carries *N* uniformly distributed elementary charges *e*, either positive or negative ones, implying an average surface charge density $\sigma_{0}=\pm eN/A$. The charges exhibit some short-range order (as is the case in a fluid); long-range order is not necessary for our model to apply. For a lipid layer, $|\sigma_{0}|≲e/{nm}^{2}$, with the exact value depending on the fraction of charged lipids. When $\sigma_{0}$ is sufficiently small, linearized Poisson–Boltzmann theory—also known as the Debye-Hückel limit—offers a simple and thus commonly used model to describe properties of the electric double layer such as the electrostatic surface potential $\Phi_{0}=\sigma_{0}l_{D}/\left(\varepsilon_{0}\varepsilon_{w}\right)$ or the free energy per unit area:

(1) $$\frac{F_{0}}{A}=\frac{\sigma_{0}^{2}}{2}\;\frac{l_{D}}{\varepsilon_{w}\varepsilon_{0}}$$

of the charged interface. The validity of Equation (1) rests on a number of assumptions, including the absence of ion-ion correlations, a uniform dielectric background (of dielectric constant $\varepsilon_{w}$), and a sufficiently small and yet uniform and continuous charge density $\sigma_{0}$ at the dielectric interface. The latter assumption must break down at some point due to the discrete nature of the charges at the interface. That is, when the separation between individual charges is much larger than the Debye screening length, these charges do not interact anymore, suggesting that Equation (1) overestimates the free energy F/A that would account properly for charge discreteness. In the following, we calculate the free energy F/A for a set of uniformly distributed discrete charges at the dielectric interface and compare it with $F_{0}/A$ in Equation (1), which ignores the discrete nature of the charges.

### 2.1. Ensemble of Charged Disks

Our method to account for the discreteness of the charges on the interface invokes the consideration of Wigner–Seitz cells [40]. A Wigner–Seitz cell serves as a unit cell of cylindrical symmetry with a circular cross-section of radius *R* and corresponding lateral area $A/N=\pi R^{2}$. We identify the *z*-axis of a cylindrical coordinate system (r,ϕ,z) with the symmetry axis of the Wigner–Seitz cell and locate the plane z≡0 at the dielectric interface. The radial coordinate *r* varies in the region 0≤r≤R, from the symmetry axis to the boundary of the unit cell. Note that the cylindrical symmetry of the unit cell renders all system properties invariant with respect to the azimuthal angle ϕ.

**Figure 1:**
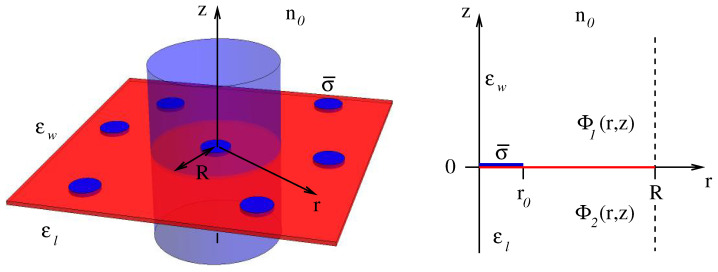
Left side: Charged circular regions (blue disks), each carrying a uniform surface charge density $\bar{\sigma }$, located at an interface (the red plane) that separates two media with dielectric constants $\varepsilon_{w}$ and $\varepsilon_{l}$ from each other. The medium with $\varepsilon_{w}$ contains a symmetric 1:1 salt solution of bulk concentration $n_{0}$. The blue transparent cylinder of radius *R* represents a unit cell (the Wigner–Seitz cell). Right side: The unit cell is fully characterized by the *r* and *z* directions of cylindrical coordinates with 0≤r≤R and −∞<z<∞. We denote the electrostatic potential for z≥0 by $\Phi_{1}\left(r,z\right)$ and for z≤0 by $\Phi_{2}\left(r,z\right)$.

If the charge on the dielectric interface was continuous and distributed uniformly, the surface charge density $\sigma \left(r\right)=\sigma_{0}$ along the radial direction would be strictly constant. In contrast, we shall consider a non-uniform, step-like surface charge density:

(2) $$\sigma \left(r\right)=\{\begin{array}{ccc} \bar{\sigma } & \mathrm{if} & r\leq r_{0}\\ 0 & \mathrm{if} & r_{0}<r\leq R, \end{array}$$

with $\bar{\sigma }=\sigma_{0}R^{2}/r_{0}^{2}$. That is, we condense all charge, $\sigma_{0}\pi R^{2}$, contained in the unit cell into a concentric circular region of radius $r_{0}$, leaving the outer region $r_{0}<r\leq R$ of the dielectric interface inside the unit cell uncharged. We are free to choose both $r_{0}$ and the total amount of charge in the unit cell $\sigma_{0}\pi R^{2}$. Yet, in order to study the influence of charge discreteness, we will eventually identify $\sigma_{0}\pi R^{2}=\bar{\sigma }\pi r_{0}^{2}$ with a single elementary charge *e* and consider the limit $r_{0}\to 0$ of a point charge. Our goal is to calculate the corresponding Debye-Hückel free energy $F(r_{0})$ in that limit. The left side of Figure 1 shows an illustration of the dielectric interface (red plane) with seven circular disks of radius $r_{0}$ (shown in blue color) that carry a charge density $\bar{\sigma }$ each, one with its unit cell (the Wigner–Seitz cell, marked by the blue transparent cylinder) explicitly shown.

The right side of Figure 1 presents the cylindrical coordinate system associated with the unit cell; the outer boundary, at r=R, is marked by a dashed line, and the interface is located at the plane z≡0.

**Figure 2:**
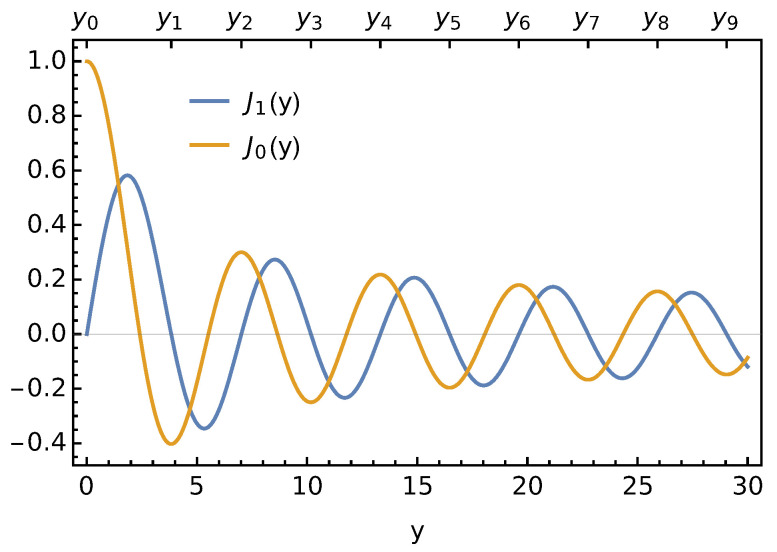
The Bessel function of the first kind and first order $J_{1}\left(y\right)$ and the Bessel function of the first kind and zeroth order $J_{0}\left(y\right)$. The $y_{n}$’s indicate the solutions of $J_{1}\left(y_{n}\right)=0$ in increasing order starting with $y_{0}=0$.

We denote the electrostatic potential for z≥0 by $\Phi_{1}\left(r,z\right)$. Due to the presence of salt, $\Phi_{1}$ fulfills the Debye-Hückel equation $l_{D}^{2}\nabla^{2}\Phi_{1}=\Phi_{1}$, where $\nabla^{2}$ denotes the Laplacian operator. Similarly, we denote the electrostatic potential for z≤0 by $\Phi_{2}\left(r,z\right)$. The potential $\Phi_{2}$ fulfills the Laplace equation $\nabla^{2}\Phi_{2}=0$. The Debye-Hückel and Laplace equations for a cylindrically symmetric unit cell and the structure of their solution can be written as:

(3) $$\begin{array}{ccc} \frac{1}{r}\frac{\partial }{\partial r}\left(r\frac{\partial \Phi_{1}}{\partial r}\right)+\frac{\partial^{2}\Phi_{1}}{\partial z^{2}}=\frac{\Phi_{1}}{l_{D}^{2}}\; & \to & \;\Phi_{1}\left(r,z\right)=\sum_{n=0}^{\infty }a_{n}\;J_{0}\left(y_{n}\frac{r}{R}\right)\;e^{-z\sqrt{{\left(\frac{y_{n}}{R}\right)}^{2}+\frac{1}{l_{D}^{2}}}}\\ \frac{1}{r}\frac{\partial }{\partial r}\left(r\frac{\partial \Phi_{2}}{\partial r}\right)+\frac{\partial^{2}\Phi_{2}}{\partial z^{2}}=0\; & \to & \;\Phi_{2}\left(r,z\right)=\sum_{n=0}^{\infty }a_{n}\;J_{0}\left(y_{n}\frac{r}{R}\right)\;e^{z\frac{y_{n}}{R}}, \end{array}$$

where $J_{0}$ denotes the Bessel function of the first kind and zeroth order. The sets of the yet undetermined constants $a_{n}$ in the expressions for $\Phi_{1}\left(r,z\right)$ and $\Phi_{2}\left(r,z\right)$ are identical to ensure the electrostatic potential remains continuous when passing through the dielectric interface at z=0. In addition, the solutions do not diverge for |z|→∞. Symmetry requires the derivatives ${\left(\partial \Phi_{1}/\partial r\right)}_{r=R}$ and ${\left(\partial \Phi_{2}/\partial r\right)}_{r=R}$ at the boundary of the unit cell to vanish. Because the Bessel function of the first kind and first order, $J_{1}\left(y\right)=-J_{0}^{\prime }\left(y\right)$, is the negative derivative of the Bessel function of the first kind and zeroth order, we demand $J_{1}\left(y_{n}\right)$ to vanish for all $y_{n}$ with integers n≥0. Hence, $y_{n}$ in Equation (3) satisfies $J_{1}\left(y_{n}\right)=0$ and is thus the n’th zero of the Bessel function of the first kind and first order. Figure 2 shows $J_{0}\left(y\right)$ and $J_{1}\left(y\right)$, with the set $y_{n}$ indicated on the upper axis for 0≤n≤9.

At the dielectric interface, the electrostatic potential must fulfill the boundary condition:

(4) $$\varepsilon_{w}{\left(\frac{\partial \Phi_{1}}{\partial z}\right)}_{z=0}-\varepsilon_{l}{\left(\frac{\partial \Phi_{2}}{\partial z}\right)}_{z=0}=-\frac{\sigma (r)}{\varepsilon_{0}}.$$

The functions $J_{0}\left(y_{n}r/R\right)$ can be used to represent a given surface charge density σ(r) through a Fourier-Bessel series $\sigma \left(r\right)=\sum_{n=0}^{\infty }\sigma_{n}J_{0}\left(y_{n}r/R\right)$ in the region 0≤r≤R, with a set of constants $\sigma_{n}$. Inserting that series and the expressions for $\Phi_{1}\left(r,z\right)$ and $\Phi_{2}\left(r,z\right)$ from Equations (3) into the boundary condition specified by Equation (4), yields the relation:

(5) $$a_{n}=\frac{1}{\varepsilon_{0}}\;\frac{\sigma_{n}}{\varepsilon_{w}\sqrt{{\left(\frac{y_{n}}{R}\right)}^{2}+\frac{1}{l_{D}^{2}}}+\varepsilon_{l}\frac{y_{n}}{R}}$$

between the coefficients $a_{n}$ and $\sigma_{n}$ for all integers n≥0. Note that, as required by symmetry, the derivative $\sigma^{\prime }\left(r\right)=-\sum_{n=1}^{\infty }\sigma_{n}\;J_{1}\left(y_{n}r/R\right)\;y_{n}/R=0$ indeed vanishes at the boundary of the unit cell. Because of $\int_{0}^{R}dr\;r\;\sigma \left(r\right)=\sum_{n=0}^{\infty }\sigma_{n}J_{1}\left(y_{n}\right)R^{2}/y_{n}=R^{2}\sigma_{0}/2$, we identify the first coefficient:

(6) $$\sigma_{0}=\frac{\int_{0}^{R}dr\;r\;\sigma \left(r\right)}{\int_{0}^{R}dr\;r\;}$$

of the Fourier-Bessel series with the average surface charge density, in agreement with how we have already used it in Equation (1). To find the remaining coefficients $\sigma_{n}$ for n≥1, we employ the orthogonality $\int_{0}^{R}dr\;r\;J_{1}\left(y_{n}r/R\right)J_{1}\left(y_{m}r/R\right)=\delta_{mn}{\left[RJ_{0}\left(y_{n}\right)\right]}^{2}/2$ of the functions $J_{1}\left(y_{n}r/R\right)$ in the region 0≤r≤R, where $\delta_{mn}$ denotes the Kronecker delta. Applying orthogonality to evaluate the integral $\int_{0}^{R}dr\;r\;\sigma^{\prime }\left(r\right)J_{1}\left(y_{n}r/R\right)$ leads to:

(7) $$\sigma_{n}=-\frac{\int_{0}^{R}dr\;r\;\sigma^{\prime }\left(r\right)J_{1}\left(y_{n}\frac{r}{R}\right)}{y_{n}\frac{R}{2}{\left[J_{0}\left(y_{n}\right)\right]}^{2}}.$$

Using the results for $a_{n}$ in Equation (5) and for $\sigma_{n}$ in Equations (6) and (7) gives rise to the explicit expression:

(8) $$\Phi_{s}\left(r\right)=\Phi_{0}-\frac{l_{D}}{\varepsilon_{0}\varepsilon_{w}}\sum_{n=1}^{\infty }\frac{J_{0}\left(y_{n}\frac{r}{R}\right)}{\sqrt{{\left(y_{n}\frac{l_{D}}{R}\right)}^{2}+1}+\frac{\varepsilon_{l}}{\varepsilon_{w}}\frac{l_{D}}{R}y_{n}}\;\frac{\int_{0}^{R}dr\;r\;\sigma^{\prime }\left(r\right)J_{1}\left(y_{n}\frac{r}{R}\right)}{y_{n}\frac{R}{2}{\left[J_{0}\left(y_{n}\right)\right]}^{2}}$$

for the surface potential $\Phi_{s}\left(r\right)=\Phi_{1}\left(r,z=0\right)=\Phi_{2}\left(r,z=0\right)$, valid for any choice of the surface charge density σ(r). Recall that $\Phi_{0}=\sigma_{0}l_{D}/\left(\varepsilon_{0}\varepsilon_{w}\right)$ is the surface potential for a strictly uniform and continuous surface charge density $\sigma_{0}$.

Our next step is to apply the general formalism of calculating the surface potential $\Phi_{s}\left(r\right)$ in Equation (8) to our specific choice of σ(r) in Equation (2). It is convenient to re-express $\sigma \left(r\right)=\bar{\sigma }u\left(r_{0}-r\right)$ in terms of the Heaviside step function u(r)=0 for r<0, u(r)=1 for r>0, and u(r)=1/2 for r=0. This implies $\sigma^{\prime }\left(r\right)=-\bar{\sigma }\delta \left(r-r_{0}\right)$, where δ(r) denotes the Dirac delta function. Hence, $\int_{0}^{R}dr\;r\;\sigma^{\prime }\left(r\right)J_{1}\left(y_{n}r/R\right)=-\bar{\sigma }r_{0}J_{1}\left(y_{n}r_{0}/R\right)$, and thus:

(9) $$\begin{array}{ccc} \frac{\sigma (r)}{\sigma_{0}} & = & 1+\sum_{n=1}^{\infty }\frac{J_{0}\left(y_{n}\frac{r}{R}\right)\;2\frac{R}{r_{0}}\;J_{1}\left(y_{n}\frac{r_{0}}{R}\right)}{y_{n}{\left[J_{0}\left(y_{n}\right)\right]}^{2}},\\ \frac{\Phi_{s}\left(r\right)}{\Phi_{0}} & = & 1+\sum_{n=1}^{\infty }\frac{J_{0}\left(y_{n}\frac{r}{R}\right)}{\sqrt{{\left(y_{n}\frac{l_{D}}{R}\right)}^{2}+1}+\frac{\varepsilon_{l}}{\varepsilon_{w}}\frac{l_{D}}{R}y_{n}}\;\frac{2\frac{R}{r_{0}}\;J_{1}\left(y_{n}\frac{r_{0}}{R}\right)}{y_{n}{\left[J_{0}\left(y_{n}\right)\right]}^{2}}. \end{array}$$

**Figure 3:**
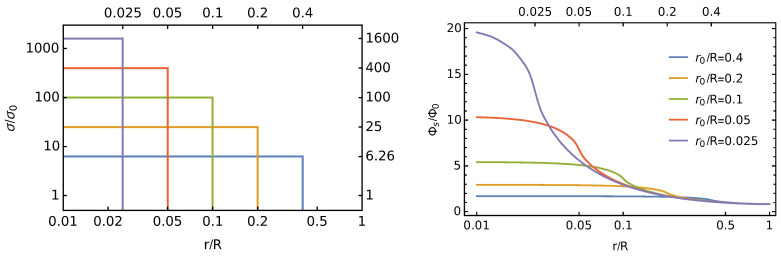
Scaled surface charge density $\sigma \left(r\right)/\sigma_{0}$ (left diagram) and scaled surface potential $\Phi_{s}\left(r\right)/\Phi_{0}$ (right diagram) according to Equation (9), with $l_{D}=R$ and $\varepsilon_{l}=\varepsilon_{w}$. In both diagrams, the different curves correspond to $r_{0}/R=0.4$ (blue), $r_{0}/R=0.2$ (orange), $r_{0}/R=0.1$ (green), $r_{0}/R=0.05$ (red), and $r_{0}/R=0.025$ (purple).

Equations (9) represent the Fourier-Bessel series of the surface charge density according to Equation (2) as well as the corresponding electrostatic surface potential $\Phi_{s}\left(r\right)$ at the dielectric interface. Recall that our choice $\bar{\sigma }=\sigma_{0}R^{2}/r_{0}^{2}$ ensures the total charge in the unit cell is $\pi R^{2}\sigma_{0}$. Both $\sigma \left(r\right)/\sigma_{0}$ and $\Phi_{s}\left(r\right)/\Phi_{0}$ are functions of r/R and depend on the relative size of the charged circular region as compared to the cell size, $r_{0}/R$. The scaled potential depends on two additional parameters, $l_{D}/R$ and $\varepsilon_{l}/\varepsilon_{w}$. Figure 3 shows $\sigma \left(r\right)/\sigma_{0}$ (left diagram) and $\Phi_{s}\left(r\right)/\Phi_{0}$ (right diagram) for different choices of $r_{0}/R$, all with $l_{D}=R$ and $\varepsilon_{l}=\varepsilon_{w}$.

In the limit $r_{0}\to 0$, the charge distribution $\sigma \left(r\right)=\sigma_{0}\pi R^{2}\delta \left(r\right)$ approaches that of a single point charge, and the corresponding surface potential becomes:

(10) $$\frac{\Phi_{s}\left(r\right)}{\Phi_{0}}=1+\sum_{n=1}^{\infty }\frac{J_{0}\left(y_{n}\frac{r}{R}\right)}{\sqrt{{\left(y_{n}\frac{l_{D}}{R}\right)}^{2}+1}+\frac{\varepsilon_{l}}{\varepsilon_{w}}\frac{l_{D}}{R}y_{n}}\;\frac{1}{{\left[J_{0}\left(y_{n}\right)\right]}^{2}}.$$

Note that $\Phi_{s}\left(r\right)$ in Equation (10) diverges at the location of the point charge, r=0.

### 2.2. Single Isolated Charged Disk

In order to calculate the interaction energy between an ensemble of charged disks (and eventually an ensemble of point charges), we will also consider a single isolated charged disk as the reference state. We denote the electrostatic surface potential produced by an isolated charged disk of radius $r_{0}$ by $\Phi_{s}^{self}\left(r\right)$. The calculation of $\Phi_{s}^{self}\left(r\right)$ proceeds analogously to that of $\Phi_{s}\left(r\right)$, yet for an infinitely large unit cell size, R→∞, while preserving $\bar{\sigma }=\sigma_{0}R^{2}/r_{0}^{2}$ as specified in Equation (2). In this case, we shall make use of a Hankel transform (also known as Bessel–Fourier transform) instead of a Bessel-Fourier series. For the surface charge density, we employ the identity:

(11) $$\sigma \left(r\right)=\int_{0}^{\infty }dk\;k\;J_{0}\left(kr\right)\;\int_{0}^{\infty }d\bar{r}\;\bar{r}\;\sigma \left(\bar{r}\right)J_{0}\left(k\bar{r}\right)$$

and make use of the electrostatic potentials:

(12) $$\Phi_{1}\left(r,z\right)=\int_{0}^{\infty }dk\;k\;a\left(k\right)\;J_{0}\left(kr\right)\;e^{-\sqrt{k^{2}+\frac{1}{l_{D}^{2}}}z},\;\Phi_{2}\left(r,z\right)=\int_{0}^{\infty }dk\;k\;a\left(k\right)\;J_{0}\left(kr\right)\;e^{kz}.$$

Inserting Equations (11) and (12) into the boundary condition specified in Equation (4) allows us to calculate the zeroth order Hankel transform:

(13) $$a\left(k\right)=\frac{1}{\varepsilon_{0}}\;\frac{\int_{0}^{\infty }dr\;r\;\sigma \left(r\right)\;J_{0}\left(kr\right)}{\varepsilon_{w}\sqrt{k^{2}+\frac{1}{l_{D}^{2}}}+\varepsilon_{l}k}=\frac{1}{\varepsilon_{0}}\;\frac{\bar{\sigma }\;r_{0}\;J_{1}\left(kr_{0}\right)}{k\left(\varepsilon_{w}\sqrt{k^{2}+\frac{1}{l_{D}^{2}}}+\varepsilon_{l}k\right)},$$

of the surface potential $\Phi_{s}^{self}\left(r\right)=\Phi_{1}\left(r,z=0\right)=\Phi_{2}\left(r,z=0\right)$, where the equality on the right-hand side of Equation (13) is based on using the surface charge density in Equation (2) with R→∞. Knowing the function a(k), introducing the dimensionalized wave number y=kR, and replacing $\bar{\sigma }=\sigma_{0}R^{2}/r_{0}^{2}$ allow us to express the scaled surface potential as:

(14) $$\frac{\Phi_{s}^{self}\left(r\right)}{\Phi_{0}}=\frac{R}{r_{0}}\int_{0}^{\infty }dy\frac{J_{0}\left(y\frac{r}{R}\right)J_{1}\left(y\frac{r_{0}}{R}\right)}{\sqrt{{\left(y\frac{l_{D}}{R}\right)}^{2}+1}+\frac{\varepsilon_{l}}{\varepsilon_{w}}\frac{l_{D}}{R}y}.$$

**Figure 4:**
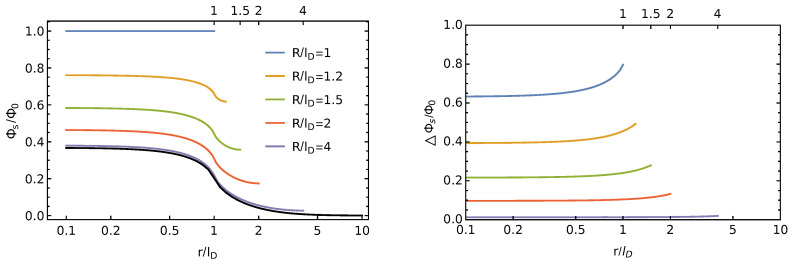
Left diagram: The scaled potential $\Phi_{s}\left(r\right)/\Phi_{0}$ for a charged disk of radius $r_{0}/l_{D}=1$ and surface charge density $\sigma_{0}$ that resides in a unit cell of radius $R/l_{D}=1$ (blue curve), $R/l_{D}=1.2$ (orange), $R/l_{D}=1.5$ (green), $R/l_{D}=2$ (red), $R/l_{D}=4$ (purple), and $R/l_{D}\to \infty$ (black). The black curve is calculated using $\Phi_{s}^{self}\left(r\right)/\Phi_{0}$ in Equation (14), and all others are based on $\Phi_{s}\left(r\right)/\Phi_{0}$ in Equation (9), with $\varepsilon_{l}=\varepsilon_{w}$ in each case. Right diagram: The difference $▵\Phi_{s}\left(r\right)/\Phi_{0}$ between each colored curve and the black curve on the left diagram. The legend on the left diagram also applies to the right diagram. Note that $▵\Phi_{s}\left(r\right)=\Phi_{s}\left(r\right)-\Phi_{s}^{self}\left(r\right)$ corresponds to the surface potential in the unit cell produced by all charged disks other than that in the considered unit cell.

Note that $\Phi_{s}\left(r\right)$ in Equation (10) applies to a circular disk of size $r_{0}$ and charge density $\bar{\sigma }$ inside a unit cell of radius *R*, whereas $\Phi_{s}^{self}\left(r\right)$ in Equation (14) describes the surface potential in the limit on an infinitely large unit cell for the same circular disk of size $r_{0}$ and charge density $\bar{\sigma }$. In both cases, *R*, $r_{0}$, and $\sigma_{0}$—which is contained in $\Phi_{0}=\sigma_{0}l_{D}/\left(\varepsilon_{0}\varepsilon_{w}\right)$—define the surface charge density $\bar{\sigma }=\sigma_{0}R^{2}/r_{0}^{2}$ of the charged disk. The left diagram of Figure 4 shows an example for $\Phi_{s}^{self}\left(r\right)/\Phi_{0}$ (black curve), calculated for $r_{0}/l_{D}=1$, $R/l_{D}=1$, and $\varepsilon_{l}=\varepsilon_{w}$. That is, the isolated charged disk described by the black curve has a radius equal to the Debye length and a surface charge density $\sigma_{0}$. Decreasing the unit cell radius from infinity to finite values (while keeping the disk unchanged) increases the scaled potential in the unit cell, $\Phi_{s}\left(r\right)/\Phi_{0}$, as shown for $R/l_{D}=4$ (purple), $R/l_{D}=2$ (red), $R/l_{D}=1.5$ (green), $R/l_{D}=1.2$ (orange), and $R/l_{D}=1$ (blue). In the final case (the blue line), the sizes of unit cell and disk are identical, $r_{0}=R=l_{D}$, implying $\Phi_{s}\left(r\right)/\Phi_{0}=1$.

The right diagram of Figure 4 shows the scaled difference potential $▵\Phi_{s}\left(r\right)/\Phi_{0}$, defined through $▵\Phi_{s}\left(r\right)=\Phi_{s}\left(r\right)-\Phi_{s}^{self}\left(r\right)$, for each displayed case in the left diagram, with the same color coding. For example, the blue curve in the right diagram displays the difference between the blue and black curves in the left diagram. Importantly, $▵\Phi_{s}\left(r\right)$ corresponds to the electrostatic potential produced by all charged disks other than the central one located in our unit cell. Below, we will use $▵\Phi_{s}\left(r\right)$ to calculate the Debye-Hückel free energy of the electric double layer at the dielectric interface.

We also point out that the limit $r_{0}\to 0$ of Equation (14) describes a single point charge that is located at the dielectric interface. The resulting scaled surface potential:

(15) $${\left(\frac{\Phi_{s}^{self}\left(r\right)}{\Phi_{0}}\right)}_{r_{0}\to 0}=\frac{1}{2}\int_{0}^{\infty }dy\;y\;\frac{J_{0}\left(y\frac{r}{R}\right)}{\sqrt{{\left(y\frac{l_{D}}{R}\right)}^{2}+1}+\frac{\varepsilon_{l}}{\varepsilon_{w}}\frac{l_{D}}{R}y}$$

was first computed by Stillinger [22] and was later extended to off-interfacial locations of the charge and to bilayer geometries [24,41,42]. Hurd [43] demonstrated that in the limit $\varepsilon_{l}\ll \varepsilon_{w}$ and $l_{D}\ll r$, Equation (15) can be expressed as:

(16) $${\left(\frac{\Phi_{s}^{self}\left(r\right)}{\Phi_{0}}\right)}_{r_{0}\to 0}=\frac{1}{2}\frac{R^{2}}{l_{D}^{2}}\;\left[\frac{l_{D}}{r}e^{-r/l_{D}}+\frac{\varepsilon_{l}}{\varepsilon_{w}}\;\frac{l_{D}^{3}}{r^{3}}\right].$$

This is the sum of a screened Coulomb and dipole potential. The latter is long-range and is expected to dominate for sufficiently small average surface charge densities $\sigma_{0}=e/\left(\pi R^{2}\right)$.

### 2.3. Debye-Hückel Free Energy

Consider *N* isolated non-interacting circular disks, each of radius $r_{0}$ and located at the dielectric interface. Assembling the disks into an array of average surface charge density $\sigma_{0}$ and radius *R* of the unit cell is associated with an electrostatic interaction free energy per unit area F/A. We calculate that energy by multiplying the surface charge density at each point in a unit cell σ(r) with the electrostatic potential produced by all other unit cells $▵\Phi_{s}\left(r\right)$,

(17) $$\frac{F}{A}=\frac{1}{2}\;\frac{\int_{0}^{R}dr\;r\;\sigma \left(r\right)▵\Phi_{s}\left(r\right)}{\int_{0}^{R}dr\;r\;},$$

where the factor 1/2 avoids double-counting of the cell-cell interactions. The free energy $F=F(r_{0})$ in Equation (17) is a function of disk size, with the limit $r_{0}\to 0$ corresponding to discrete point-like charges. In the other limit, $r_{0}=R$, all charge is uniformly spread over the surface. In this case, $F(r_{0}=R)$ corresponds to the energy cost of merging initially isolated, preformed disks (all of radius *R* and surface charge density $\sigma_{0}$) into a uniformly charged interface with surface charge density $\sigma_{0}$. Note that $F(r_{0}=R)$ would become identical to $F_{0}$ upon ignoring the self-energy of the preformed disks (that is, upon replacing $▵\Phi_{s}\left(r\right)$ by $\Phi_{s}\left(r\right)$ in Equation (17)). Using the definition of $▵\Phi_{s}\left(r\right)$, as well as the expressions for σ(r) in Equation (2), for $\Phi_{s}\left(r\right)/\Phi_{0}$ in Equation (9), and for $\Phi_{s}^{self}\left(r\right)/\Phi_{0}$ in Equation (14), and integrating over the Bessel function give rise to the scaled Debye-Hückel free energy:

(18) $$\frac{F}{F_{0}}=1+{\left(\frac{R}{r_{0}}\right)}^{2}\left\{\sum_{n=1}^{\infty }\frac{{\left[\frac{2J_{1}\left(y_{n}\frac{r_{0}}{R}\right)}{y_{n}\;J_{0}\left(y_{n}\right)}\right]}^{2}}{\sqrt{{\left(y_{n}\frac{l_{D}}{R}\right)}^{2}+1}+\frac{\varepsilon_{l}}{\varepsilon_{w}}\frac{l_{D}}{R}y_{n}}-\int_{0}^{\infty }dy\frac{\frac{2}{y}{\left[J_{1}\left(y\frac{r_{0}}{R}\right)\right]}^{2}}{\sqrt{{\left(y\frac{l_{D}}{R}\right)}^{2}+1}+\frac{\varepsilon_{l}}{\varepsilon_{w}}\frac{l_{D}}{R}y}\right\},$$

where $F_{0}$ is specified in Equation (1). Equation (18) is a major result of the present work; it is the relative change of the Debye-Hückel free energy of an ensemble of uniformly charged disks (each disk of radius $r_{0}$ in a Wigner–Seitz cell of radius *R*) as compared to the continuum limit, where due to the absence of charge discreteness, self-energies become irrelevant and where all charge is smeared out to a uniform surface charge density. This relative change is fully characterized by specifying the three ratios: $r_{0}/R$, $l_{D}/R$, and $\varepsilon_{l}/\varepsilon_{w}$. To obtain a numerical estimate of $F/F_{0}$, we carry out the summation and integration up to an upper limit only:

(19) $$\frac{F}{F_{0}}=1+{\left(\frac{R}{r_{0}}\right)}^{2}\left\{\sum_{n=1}^{n_{max}}\frac{{\left[\frac{2J_{1}\left(y_{n}\frac{r_{0}}{R}\right)}{y_{n}\;J_{0}\left(y_{n}\right)}\right]}^{2}}{\sqrt{{\left(y_{n}\frac{l_{D}}{R}\right)}^{2}+1}+\frac{\varepsilon_{l}}{\varepsilon_{w}}\frac{l_{D}}{R}y_{n}}-\int_{0}^{y_{n_{max}}}dy\frac{\frac{2}{y}{\left[J_{1}\left(y\frac{r_{0}}{R}\right)\right]}^{2}}{\sqrt{{\left(y\frac{l_{D}}{R}\right)}^{2}+1}+\frac{\varepsilon_{l}}{\varepsilon_{w}}\frac{l_{D}}{R}y}\right\}.$$

**Figure 5:**
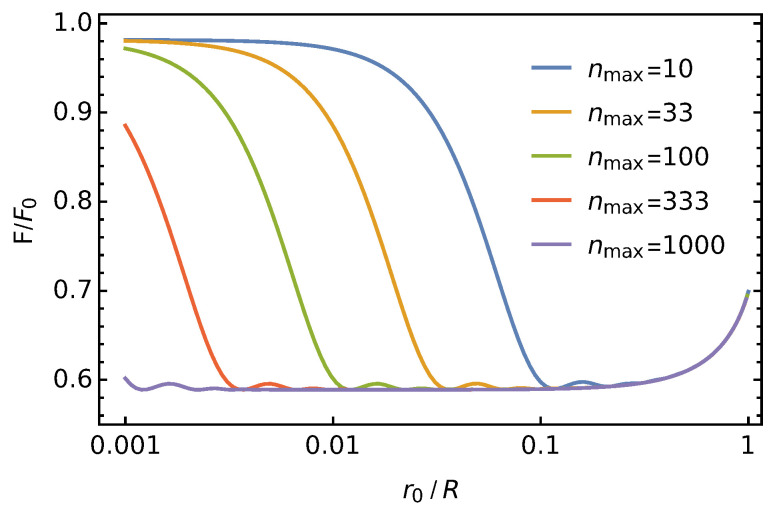
Scaled Debye-Hückel free energy $F/F_{0}$ according to Equation (19) as a function of relative disk size $r_{0}/R$ for fixed $R/l_{D}=1$ and $\varepsilon_{l}=\varepsilon_{w}$. Different curves correspond to $n_{max}=10$ (blue), $n_{max}=33$ (orange), $n_{max}=100$ (green), $n_{max}=333$ (red), $n_{max}=1000$ (purple).

Here, $y_{n_{max}}$ denotes the $n_{max}$’th zero of $J_{1}\left(y\right)$; see Figure 2. In Figure 5, we show $F/F_{0}$ as a function of $r_{0}/R$ for fixed $R/l_{D}=1$ and $\varepsilon_{l}=\varepsilon_{w}$. Different curves correspond to different $n_{max}$.

Clearly, for growing $n_{max}$, the numerical approximation for $F/F_{0}$ in Equation (19) approaches a limiting value for every non-vanishing disk size $r_{0}>0$, yet not for the point charge limit $r_{0}\to 0$. To obtain an accurate estimate even for point charges, we point out that because the derivative of $J_{1}\left(r_{0}y\right)/r_{0}$ with respect to $r_{0}$ at the position $r_{0}=0$ vanishes, the derivative:

(20) $${\left(\frac{d(F/F_{0})}{dr_{0}}\right)}_{r_{0}=0}=0$$

also vanishes. Hence, we obtain an accurate numerical value of $F/F_{0}$ in the limit $r_{0}\to 0$ by calculating $F/F_{0}$ at a sufficiently small non-vanishing position $r_{0}$ and for sufficiently large $n_{max}$. More specifically, we choose $r_{0}\ll \min\left(R,l_{D}\right)$ and $n_{max}\gg R/r_{0}$. The example shown in Figure 5 applies to $R=l_{D}$, for which $r_{0}≲0.1R$ and $n_{max}≳3R/r_{0}$ are convenient choices. We will use that method instead of the formal limit of Equation (18),

(21) $$\lim_{r_{0}\to 0}\frac{F}{F_{0}}=1+\sum_{n=1}^{\infty }\frac{1}{\sqrt{{\left(y_{n}\frac{l_{D}}{R}\right)}^{2}+1}+\frac{\varepsilon_{l}}{\varepsilon_{w}}\frac{l_{D}}{R}y_{n}}\;\frac{1}{{\left[J_{0}\left(y_{n}\right)\right]}^{2}}-\int_{0}^{\infty }dy\frac{y/2}{\sqrt{{\left(y\frac{l_{D}}{R}\right)}^{2}+1}+\frac{\varepsilon_{l}}{\varepsilon_{w}}\frac{l_{D}}{R}y},$$

which contains two diverging contributions and thus is unsuitable to be computed numerically.

**Figure 6:**
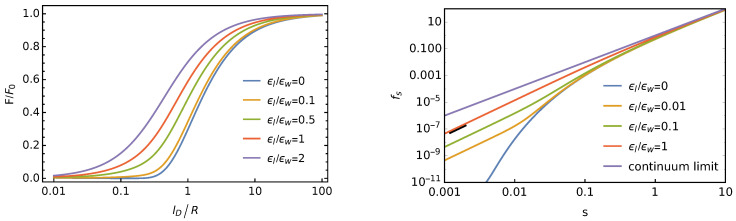
Left diagram: Scaled Debye-Hückel free energy $F/F_{0}$ for an ensemble of discrete point charges as a function of the scaled Debye length $l_{D}/R$, with $R=\sqrt{e/(\sigma_{0}\pi )}$ being the radius of the Wigner–Seitz cell. Different curves correspond to $\varepsilon_{l}/\varepsilon_{w}=0$ (blue), $\varepsilon_{l}/\varepsilon_{w}=0.1$ (orange), $\varepsilon_{l}/\varepsilon_{w}=0.5$ (green), $\varepsilon_{l}/\varepsilon_{w}=1$ (red), and $\varepsilon_{l}/\varepsilon_{w}=2$ (purple). Right diagram: The scaled free energy $f_{s}=2l_{D}^{3}\varepsilon_{w}\varepsilon_{0}F/\left(e^{2}A\right)$ as a function of the scaled surface charge density $s=l_{D}^{2}\times \left(\sigma_{0}/e\right)$ for $\varepsilon_{l}/\varepsilon_{w}=0$ (blue), $\varepsilon_{l}/\varepsilon_{w}=0.01$ (orange), $\varepsilon_{l}/\varepsilon_{w}=0.1$ (green), and $\varepsilon_{l}/\varepsilon_{w}=1$ (red). The uppermost straight line (shown in purple) corresponds to the continuum limit $f_{s}=s^{2}$ where the discreteness of the charges is ignored (see also Equation (1)). The short black line segment close to the red line is a guide to the eye; it has a slope of 2.5 and thus indicates a behavior $f_{s}∼s^{2.5}$, as observed for non-vanishing $\varepsilon_{l}/\varepsilon_{w}$ in the limit of small *s*.

A this point, we have acquired the ability to numerically compute the Debye-Hückel free energy of a planar dielectric interface with an ensemble of discrete point charges (given by Equation (21), yet calculated using Equation (19) for sufficiently large $n_{max}$ and small $r_{0}/R$). The influence of considering discrete charges versus a smeared uniform charge density is directly reflected by the ratio $F/F_{0}$. This ratio depends only on the two parameters $l_{D}/R$ and $\varepsilon_{l}/\varepsilon_{w}$. The left diagram of Figure 6 shows how $F/F_{0}$ varies as a function of $l_{D}/R$.

The cell radius $R=\sqrt{e/(\sigma_{0}\pi )}$ must be chosen such that one elementary charge $e=\sigma_{0}\pi R^{2}$ is contained inside a single unit cell. Different curves correspond to different $\varepsilon_{l}/\varepsilon_{w}$. The ratio approaches $F/F_{0}=0$ for sufficiently small $l_{D}$ and $F/F_{0}=1$ for sufficiently large $l_{D}$. Indeed, while for very small $l_{D}$, the individual charges are screened and thus do not significantly interact with each other, the discreteness of the charge becomes irrelevant at large Debye lengths, implying that *F* and $F_{0}$ become identical. Note also that $F/F_{0}$ increases with growing $\varepsilon_{l}$. Recall that *F* represents the interaction energy of point charges that are initially separated on the dielectric interface and are then brought together to form an ensemble of average surface charge density $\sigma_{0}$. When $\varepsilon_{l}=0$, the charges are only able to interact through the salt-containing medium, which eliminates long-range interactions. For $\varepsilon_{l}>0$, an electric field also forms in the salt-free medium. This field is long-range and results from the dipole that each surface charge forms with its associated diffuse counterion cloud; see Equation (16). These additional long-range interactions cause $F/F_{0}$ to increase with growing $\varepsilon_{l}$.

When changing the surface charge density $\sigma_{0}$ at fixed Debye length $l_{D}$, it is convenient to plot the scaled free energy $f_{s}=2l_{D}^{3}\varepsilon_{w}\varepsilon_{0}F/\left(e^{2}A\right)$ as a function of the scaled surface charge density $s=l_{D}^{2}\times \left(\sigma_{0}/e\right)$. The right diagram of Figure 6 shows a double-logarithmic plot of $f_{s}$ as a function of *s*, for different ratios of $\varepsilon_{l}/\varepsilon_{w}$. For the continuum model (see Equation (1), where the discrete nature of the charges is neglected), $f_{s}=s^{2}$, as indicated by the uppermost curve in purple (which has a slope of two in the double-logarithmic plot). The other curves correspond to different ratios of $\varepsilon_{l}/\varepsilon_{w}$. The lowest one, for $\varepsilon_{l}/\varepsilon_{w}=0$ (blue color), differs substantially from the second lowest one, derived for $\varepsilon_{l}/\varepsilon_{w}=0.01$ (orange color). Indeed, the absence of long-range interactions for $\varepsilon_{l}/\varepsilon_{w}=0$ causes $f_{s}$ to decrease exponentially, as dictated by the screened Coulomb interaction. For non-vanishing $\varepsilon_{l}/\varepsilon_{w}$, the charges on the dielectric interface exhibit dipolar interactions, which leads to the scaled free energy $f_{s}∼s^{5/2}$. This follows immediately from $F/A∼\left(N/A\right)\times 1/r^{3}$, as well as $N/A∼\sigma_{0}$ and $1/r^{2}∼\sigma_{0}$ plus the definitions of $f_{s}$ and *s*. Hence, we expect all curves with $\varepsilon_{l}/\varepsilon_{w}>0$ in the right diagram of Figure 6 to adopt a slope of 2.5 for a sufficiently small scaled average surface charge density *s*. This is exactly what we observe as indicated by the short black line segment, which has a slope of 2.5. The change from $f_{s}∼s^{2}$ to $f_{s}∼s^{2.5}$ as a function of decreasing *s* is the most decisive feature of the Debye-Hückel model for discrete surface charges at a dielectric interface. Its accurate quantification is the main achievement of the present work.

## 3. Conclusions

Charges are often located at dielectric interfaces between a salt-containing aqueous solution and a salt-free medium of low dielectric constant. When the surface density of the charges becomes sufficiently small, their discrete nature cannot be ignored. We derived a free energy expression (see Equation (21)) that is valid within the linear Debye-Hückel framework for an ensemble of discrete charges at a planar dielectric interface. When the charge-to-charge distance on the interface is much smaller than the Debye length, we recover the well-known Debye-Hückel free energy expression for a continuous charge distribution, with the free energy $F∼\sigma_{0}^{2}$ being proportional to the square of the surface charge density $\sigma_{0}$. When the charge-to-charge distance on the interface becomes much larger than the Debye length, interactions between dipoles formed by the interfacial charges with their counterions in solution become dominant, changing the free energy to a relationship $F∼\sigma_{0}^{2.5}$. Biological cells operate at a Debye length of $l_{D}\approx 1$ nm. The nearest-neighbor distances between charges on biomolecular aggregates are often significantly larger than the Debye length. For example, the nearest-neighbor distance between the charges on a lipid membrane with 10% anionic lipids is about three times larger than the Debye length, necessitating accounting for the discreteness of the charge when using free energies to model membrane properties, such as domain formation, bending stiffness, spinodal decomposition, charge reversal [44,45], and renormalization [46], or differential capacitance [47,48]. Our present work may assist in this kind of modeling.

We emphasize that our model is valid within the Debye-Hückel limit, where correlations and ion size effects are neglected. Applying Poisson–Boltzmann theory or other non-linear approaches that account for finite ion sizes to discrete charges at a dielectric interface will likely not yield analytical expressions, but can be implemented numerically.
